# Cost-effectiveness of two screening strategies based on Chinese diabetes risk score for pre-diabetes in China

**DOI:** 10.3389/fpubh.2022.1018084

**Published:** 2022-11-30

**Authors:** Jingjing Hao, Qiang Yao, Yidie Lin, Yue Sun, Baiyang Zhang, Meijing Hu, Jing Zhang, Ningxuan Zhao, Jiao Pei, Zhonghua Liu, Cairong Zhu

**Affiliations:** ^1^Department of Epidemiology and Health Statistics, West China School of Public Health and West China Fourth Hospital, Sichuan University, Chengdu, China; ^2^Department of Cardiology, Daping Hospital, The Third Military Medical University, Chongqing, China; ^3^West China School of Public Health and West China Fourth Hospital, Sichuan University, Chengdu, China

**Keywords:** screening, Chinese diabetes risk score, pre-diabetes, Markov, cost-effectiveness

## Abstract

**Objective:**

Studies have shown that screening for pre-diabetes mellitus (pre-DM) is essential to prevent type 2 diabetes mellitus (T2DM). This study evaluates the cost-effectiveness of two screening strategies that apply the Chinese Diabetes Risk Score (CDRS) to screen for pre-DM in China.

**Methods:**

A Markov microsimulation model was conducted from a social perspective, and the input parameters were obtained from published literature or publicly available data. Two screening strategies for pre-DM based on CDRS were built and compared with the control group to determine the cost-effective strategy. The screening strategy of the control group was screening for pre-DM by fasting plasma glucose (FPG) test in adults undergoing annual health examination and no screening in adults without an annual health examination (status quo). Two screening strategies were strategy 1: screening for pre-DM using CDRS in all adults (including with or without an annual health examination); and strategy 2: supplemental self-screening for pre-DM using CDRS in adults without an annual health examination, based on the status quo. We focus on the cumulative prevalence of T2DM and the incremental cost-effectiveness ratio which signifies the cost per case of T2DM prevented. We also evaluated the cost-effectiveness from the health system perspective. One-way and probabilistic sensitivity analyses were conducted to verify the robustness of the results.

**Results:**

The costs a case of T2DM prevented for strategy 1 compared with the control group and strategy 2 were $299.67 (95% CI 298.88, 300.46) and $385.89 (95% CI 381.58, 390.20), respectively. In addition, compared with the control group, the cost of strategy 2 to prevent a case of T2DM was $272.23 (95% CI 271.50, 272.96).

**Conclusions:**

Screening for pre-DM using CDRS in all adults was the most cost-effective health policy. We suggest that medical institutions replace FPG with CDRS for pre-DM screening; at the same time, self-screening for pre-DM using CDRS is widely promoted among adults without an annual health examination. There were still some disputes about how CDRS is included in the health examination projects, so strategy 2 should be considered as an alternative screening strategy. Findings provide a reference for the application of the CDRS in pre-DM screening and contribute to T2DM prevention.

## Introduction

Type 2 diabetes mellitus (T2DM) poses huge health and economic burden in China. The prevalence of T2DM, including diagnosed and undiagnosed, was 10.9% (1). In addition, Chinese total T2DM-related health expenditure amounted to $165.3 billion in 2021, ranking second in the world (2). Pre-diabetes mellitus (pre-DM), a transitional stage before the onset of T2DM, indicates a higher risk of developing T2DM in the future (3, 4) and denotes an already heightened risk of diabetes-related complications (5, 6). Lifestyle intervention for pre-DM has been found to delay or prevent T2DM (7–9). In China, nearly one-third of adults have pre-DM (1). Early detection of pre-DM followed by lifestyle intervention to prevent T2DM and reduce the financial burden of complications is essential.

Currently, there are invasive methods for pre-DM screening, including tests of plasma glucose and glycated hemoglobin (HbA1c), and non-invasive methods (3). However, HbA1c has still been used less frequently since it was recommended firstly by the American Diabetes Association (ADA) as an option for pre-DM or T2DM screening in 2010 (10). In China, HbA1c lacks an appropriate cut-off point and has not been standardized (11–13). Moreover, it is also not practical for screening due to the relatively high cost (13).

At present, pre-DM were found by fasting plasma glucose (FPG) test during periodic health examination (i.e., annual health examination) in China (1, 11, 14). About 58% of adults didn't have an annual health examination (15). Moreover, the main objective of the FPG test in the annual health examination is to detect T2DM. Therefore, an oral glucose tolerance test (OGTT) was further conducted if FPG≥6.1 mmol/L, resulting in many pre-diabetic patients missing (12). The 2007–2008 National T2DM Epidemiology Survey found that 71% of isolated impaired glucose tolerance (i-IGT) and 12% of impaired fasting glucose and IGT (IFG + IGT) individuals would be missed if only be detected by FPG (16).

Chinese Diabetes Risk Score (CDRS, Supplementary Table 1) is an effective non-invasive screening tool for pre-DM. CDRS was developed by Ji et al. in 2013 and included in *Guideline for the prevention and treatment of T2DM in China* as a diabetes screening tool in 2017 (17, 18). In addition, the screening strategy for pre-DM that CDRS followed by OGTT has reached expert consensus in 2020 but is not widely used (11). The sensitivity for the pre-DM diagnosis of CDRS has reached 73.4%, higher than that of FPG with a cutoff value of 6.1 mmol/L (19, 20). Compared with FPG test, CDRS screening for pre-DM may lead more normal glucose tolerance (NGT) people to get further OGTT and thus increase the cost of screening because of its lower specificity (19, 20). It is uncertain whether CDRS screening for pre-DM would be economical in China, a developing country with limited health resources, limiting its application. To our knowledge, no study analyzed the cost-effectiveness of the pre-DM screening strategy using CDRS in China. Therefore, two screening strategies for pre-DM based on CDRS were built and compared with the status quo of finding pre-DM to determine the cost-effective strategy, providing evidence for the application of CDRS in pre-DM screening.

## Methods

### Screening and intervention strategy

**Figure 1:**
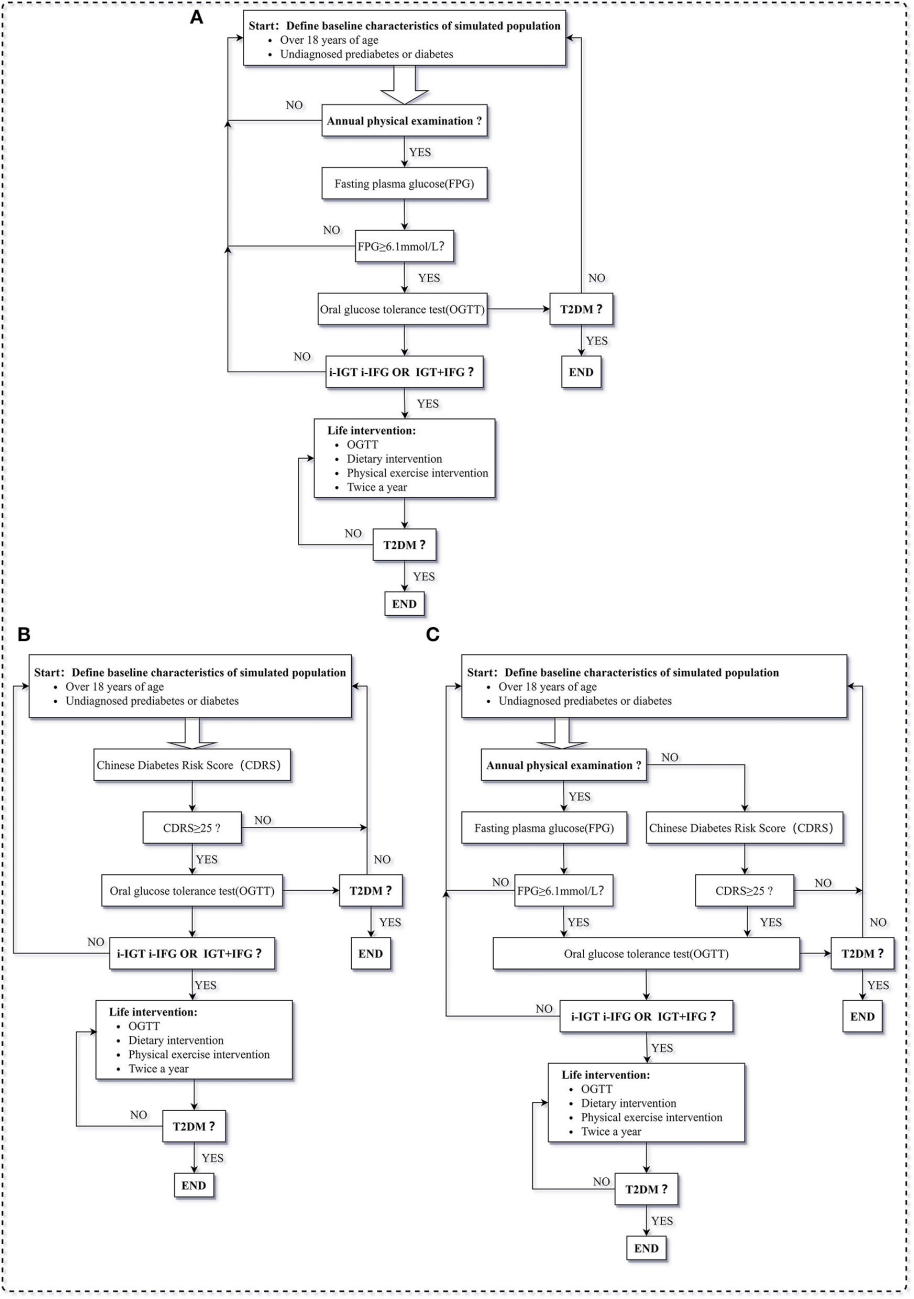
Description of steps to pre-DM screening strategies, **(A)** is screening strategy of control group, **(B)** is screening strategy of screening strategy 1, **(C)** is screening strategy of screening strategy 2. T2DM, type 2 diabetes mellitus; FPG, fasting glucose test; I-IFG, isolated impaired fasting glucose; I-IGT, isolated impaired glucose tolerance; IFG + IGT, impaired fasting glucose and impaired glucose tolerance; NGT, normal glucose tolerance; OGTT, oral glucose tolerance test; Pre-DM, pre-diabetes mellitus; CDRS, Chinese Diabetes Risk Score.

The screening strategy of the control group was the status quo that screening for pre-DM using FPG test in adults undergoing annual health examination and no screening in adults without annual health examination (Figure 1A). The screening strategy 1 was that screen for pre-DM using CDRS in all adults, including with or without annual health examination (Figure 1B). However, CDRS is not fully included in the annual health examination system in China (21). Some medical institutions cover CDRS as one of the payment items in the health examination package (21). CDRS is a questionnaire that includes common questions such as age, sex, BMI, blood pressure, waist circumference, and family history of diabetes. It does not require complex medical examinations and can be completed without the guidance of doctors (17). CDRS itself is not costly, which contradicts the need for medical institutions to make profits (22). There are still many disputes about whether CDRS is included in the health examination projects in conventional payment, voluntary payment, or without charge. Therefore, we also constructed screening strategy 2: based on the status quo, supplemental self-screening for pre-DM using CDRS in adults without annual health examination (Figure 1C).

The pre-DM screening strategies proceeded in a stepwise manner and were conducted annually. If the preliminary test indicated that the patients might be pre-DM, it requested a further OGTT for diagnosis. Once diagnosed with pre-DM, a series of lifestyle interventions would be conducted. Lifestyle interventions were individual consultations on daily food intake and physical activity by community general practitioners. They quantify food intake and physical activity by standardized forms and interviews, and provide individualized recommendations (23). It was set to be twice a year until patients change their health status (12).

### Study design

**Figure 2:**
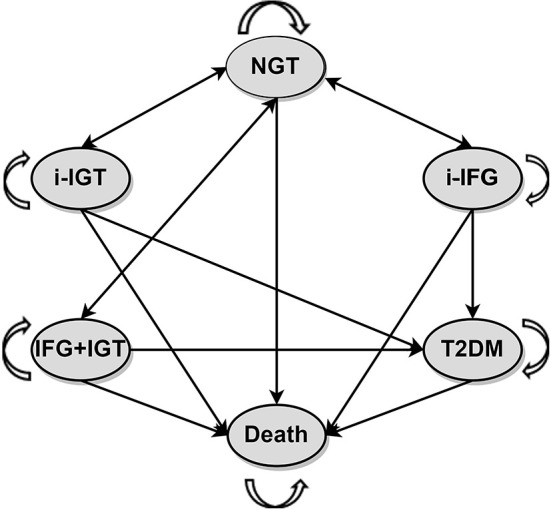
Markov state-transition model with the six health states. T2DM, type 2 diabetes mellitus; I-IFG, isolated impaired fasting glucose; I-IGT, isolated impaired glucose tolerance; IFG + IGT, impaired fasting glucose and impaired glucose tolerance; NGT, normal glucose tolerance.

Since there is no universally implemented CDRS screening for pre-DM, we used a Markov microsimulation model to estimate disease progression, cost and effectiveness of different screening strategies. The Markov model consisted of six health states: NGT, isolated impaired fasting glucose (i-IFG), i-IGT, IFG + IGT, T2DM, and death (Figure 2). Diagnosis of pre-DM and T2DM was made using OGTT, as recommended by the ADA in 2021 (3). The final state, death, refers to all-cause mortality by age and sex.

In the Markov model, the number of people in each initial state was determined by the prevalence of different pre-DM and T2DM in the simulation population. Subjects with NGT may stay in this state or progress to pre-DM but cannot directly progress to the T2DM state. Pre-diabetic patients can return to the NGT state, but once they were diagnosed with T2DM, their disease course cannot be reversed. People in every health state may die for various reasons and drop out of the model.

The Markov chain Monte Carlo simulation method was used to estimate the disease process of 10 million adults (18 years and older) who have not previously been diagnosed with T2DM or pre-DM. According to the average life expectancy in China, the termination condition of the Markov model was set to be 80 years old, and the model cycle was 1 year (24). We mainly focused on the cumulative prevalence of T2DM and the incremental cost-effectiveness ratio (ICER) which signifies the cost per case of T2DM prevented. The ICER was compared to the willingness-to-pay (WTP) threshold set at $12551 (the annual per-capita GDP for China in 2021).

### Input parameter of model

The input parameters of the model are shown in Table 1. Each simulated individual had a random initial age and gender, with an average age of 45.70 and females accounting for 48.92%, according to China Statistical Yearbook 2021 (24). The rate of annual health examination came from a 2018 study involving 90,208 people from 33 health examination institutions across the country (15). In addition, the prevalence of pre-DM and T2DM in this study was based on a national cross-sectional study and two extensive surveys (1, 25, 26). The sensitivity and specificity of FPG and CDRS for pre-DM and T2DM were derived from three cross-sectional surveys (19, 20, 27).

**Table 1:** Baseline values of input parameters used in models.

**Parameters**	**Expected values**	**Ranges**	**References**
**Demographic variables**			
Proportion of physical examination	42.00%	31.5–52.50%	(15)
**Prevalence of undiagnosed T2DM^a^**			
< 40	4.56%	4.15–4.97%	(1)
40–60	8.22%	7.90–8.54%	(1)
>60	12.50%	11.84–13.16%	(1)
**Prevalence of undiagnosed i-IFG^a^**			
< 40	17.28%	12.96–21.60%	(1, 25, 26)
40–60	10.18%	7.64–12.73%	(1, 25, 26)
>60	13.94%	10.45–17.42%	(1, 25, 26)
**Prevalence of undiagnosed i-IGT^a^**			
< 40	8.21%	6.16–10.26%	(1, 25, 26)
40–60	20.51%	15.38–25.64%	(1, 25, 26)
>60	22.59%	16.94–28.23%	(1, 25, 26)
**Prevalence of undiagnosed IFG + IGT^a^**			
< 40	3.72%	2.79–4.64%	(1, 25, 26)
40–60	10.93%	8.20–13.66%	(1, 25, 26)
>60	13.70%	10.27–17.12%	(1, 25, 26)
**Test efficiency**			
Sensitivity of FPG for T2DM	73.42%	55.06–91.77%	(39)
Sensitivity of FPG for i-IFG	46.09%	34.57–57.61%	(39)
Sensitivity of FPG for IFG + IGT	69.74%	52.31–87.18%	(39)
Sensitivity of CDRS for T2DM	87.60%	65.7–1.00%	(38)
Sensitivity of CDRS for Pre-DM	73.39%	55.04–91.73%	(20)
Misdiagnosis rate of CDRS for NGT	44.74%	33.55–55.92%	(20)
**Transition probabilities**			
NGT to i-IFG	2.04%	1.53–2.55%	(29)
NGT to i-IGT	6.46%	4.85–8.08%	(29)
NGT to IFG + IGT	2.76%	2.07–3.45%	(29)
**Undiagnosed i-IFG to T2DM^a^**			
< 40	7.79%	5.84–9.74%	(30)
40–50	2.90%	2.48–4.92%	(30, 33)
50–60	2.98%	1.35–2.25%	(30, 33)
>60	3.59%	2.69–4.49%	(30)
**Diagnosed i-IFG to T2DM^a^**			
< 40	9.11%	3.90–21.34%	(30, 35)
40–50	3.39%	1.45–7.93%	(30, 33, 35)
50–60	3.49%	1.49–8.17%	(30, 33, 35)
>60	4.20%	1.80–9.84%	(30, 35)
**Undiagnosed i-IGT to T2DM^a^**			
< 40	10.91%	8.18–13.64%	(30)
40–50	6.38%	5.46–10.84%	(30, 33)
50–60	6.57%	4.47–9.09%	(30, 33)
>60	7.91%	5.93–9.89%	(30)
**Diagnosed i-IGT to T2DM^a^**			
< 40	5.89%	3.93–8.84%	(30, 34)
40–50	3.44%	2.30–5.17%	(30, 33, 34)
50–60	3.55%	2.37–5.32%	(30, 33, 34)
>60	4.27%	2.85–6.41%	(30, 34)
**Undiagnosed IGT+IFG to T2DM^a^**			
< 40	8.54%	6.41–10.68%	(30)
40–50	11.11%	9.05–18.88%	(30, 33)
50–60	11.45%	7.79–15.84%	(30, 33)
>60	13.78%	10.34–17.23%	(30)
**Diagnosed IGT+IFG to T2DM^a^**			
< 40	4.27%	3.07–5.89%	(30, 34)
40–50	5.56%	4.00–7.67%	(30, 33, 34)
50–60	5.72%	4.12–7.90%	(30, 33, 34)
>60	6.89%	4.96–9.51%	(30, 34)
i-IFG to NGT	6.89%	5.16–8.61%	(36)
i-IGT to NGT	8.83%	6.62–11.03%	(36)
IGT+IFG to NGT	5.34%	4.01–6.68%	(36)
All–cause mortality	Life table	–	(37)
**Costs ($)^b^**			
FPG per event	5.49	3.64–7.97	(38–42)
OGTT per event	13.36	11.62–15.82	(38–42)
Life intervention per event	26.66	22.06–31.98	(38–42)

a These input parameters were varied by age.

b All cost data were shown in the 2021 US dollar ($1 = ¥6.45).

T2DM, type 2 diabetes mellitus; FPG, fasting glucose test; I-IFG, isolated impaired fasting glucose; I-IGT, isolated impaired glucose tolerance; IFG + IGT, impaired fasting glucose and impaired glucose tolerance; NGT, normal glucose tolerance; OGTT, oral glucose tolerance test; Pre-DM, pre-diabetes mellitus; CDRS, Chinese Diabetes Risk Score.

### Transition probability

We converted the cumulative prevalence (*P*_*t*_) obtained from published articles into annual probabilities (*P*) as the transition probability by the following equation: $P=1-e^{\left(\ln\left(1-P_{t}\right)/t\right)}\;$(28). The probability was from multi-source, including nationally representative large cohorts and local cohorts with a relatively long period of follow-up. The transition probability from NGT to different pre-DM was obtained from a 3-year community cohort study in Sichuan (29). The transition probability from pre-DM to T2DM is related to whether the patient is diagnosed. For example, a patient with i-IGT was identified and received lifestyle interventions. Consequently, it will slow their progression to the development of T2DM. The transition probability from undiagnosed pre-DM to T2DM referred to a 3-year follow-up survey, which collected health data from 25657 community residents aged 40 and above from 25 centers in China from 2011 to 2012 (30–33). The OR values of probability between different age groups were used to obtain the age-varying transition probability from pre-DM to T2DM. The transition probability from diagnosed i-IGT and IFG + IGT to T2DM was calculated according to the HR value of the lifestyle intervention group relative to the non-intervention group in the Daqing study (34). As there is no relevant study on transition probability from diagnosed i-IFG to T2DM in China, we referred to a study in Japan and conducted a sensitivity analysis (35). The transition probability from pre-DM to NGT was based on a 5-year cohort study in Shanghai (36). Age- and sex-specific mortality probability were obtained from China Health Statistics Yearbook 2020 (37).

### Economic variables

All costs of pre-DM screening and lifestyle intervention were collected from a social perspective, including direct medical costs, direct non-medical costs and indirect costs (Supplementary Table 5). Direct medical costs of screening involved the costs of laboratory examination and medical staff's time (38–40). The direct medical costs of lifestyle interventions included the costs of regular diagnostic tests, and the time costs paid by doctors with one-on-one visits (38, 39, 41). In contrast, the direct non-medical costs referred to transportation costs for doctor visits. The indirect costs, which were time costs, incorporate time spent visiting doctors and waiting time, calculated by the population average hourly wage (42). We assume there is no cost to CDRS because it is a questionnaire that includes common questions such as age, sex, BMI, blood pressure, waist circumference, and family history of diabetes. It does not require complex medical examinations and a lengthy investigation (17).

All costs were converted into US dollars at an exchange rate of $1.0 = ¥6.45 (2021). All costs were calculated using a 5% discount rate and set discount rate to 0~8% for sensitivity analysis.

### Sensitivity analysis

We reset the costs and evaluated the cost-effectiveness of several screening strategies from the health system perspective (Supplementary Table 6). One-way and probabilistic sensitivity analyses were conducted to verify the stability of the results. Sensitivity analyses were performed on all variables associated with disease prevalence, screening effectiveness, transition probability between states, costs, and discount rate. When there was no available data range, 75–125% of the initial parameters were taken as the variation range of the parameters for sensitivity analysis (43). Furthermore, the screening compliance rate is essential, and we assume it is 100% in the base model. Sensitivity analyses were also performed for compliance rates from 100% to 1%. Probabilistic sensitivity analysis considered the influence of multiple parameter changes on the results. All parameters were set to probability distributions for 1,000 iterations. Transition probabilities and costs were set as beta and lognormal distribution, respectively (44, 45). Other parameters that cannot be obtained from studies or practice were set as uniform distribution (Supplementary Methods).

## Results

### Base case analysis

The clinical and economic results were shown in Table 2. Compared with the control group, the cost a case of T2DM prevented was $299.67 (95% CI 298.88, 300.46) for strategy 1 and $272.23 (95% CI 271.50, 272.96) for strategy 2, within the WTP threshold. In addition, compared with strategy 2, the ICER of strategy 1 was $385.89 (95% CI 381.58, 390.20), which is lower than the WTP threshold.

**Table 2:** Clinical and economic results for different screening strategies.

	**Control (95% CI)**	**Strategy 1 (95% CI)**	**Strategy 2 (95% CI)**
Cost ($)	6.04 (6.04, 6.05)	46.67 (46.65, 46.69)	33.14 (33.12, 33.16)
Cumulative prevalence of T2DM (%)	63.72 (63.69, 63.74)	50.14 (50.10, 50.17)	53.75 (53.71, 53.78)
Cost per case prevented^a^	–	**299.67 (298.88, 300.46)**	**272.23 (271.50, 272.96)**
Cost per case prevented^b^	–	**385.89 (381.58, 390.20)**	**–**

a ICER values of the two screening strategies compared with the control group.

b ICER value of comparison between the two screening strategies.

T2DM, type 2 diabetes mellitus. The bold values indicate the ICER values which we are most concerned about.

The cumulative prevalence of T2DM in the control group was 63.72%; in contrast, strategies 1 and 2 showed potential effectiveness. Compared with the control group, the cumulative prevalence rates of T2DM in strategy 1 and strategy 2 were reduced by 13.58% and 9.97 %, respectively. The per capita per lifetime costs of screening were $46.67 (95% CI 46.65, 46.69) and $33.14 (95% CI 33.12, 33.16) for the strategy 1 and 2, respectively. Both screening strategy 1 and strategy 2 cost more financially than the control group.

Under strategy 1, more T2DM was prevented than in strategy 2 because of different screening methods in the adults who participate in an annual health examination. The screening method for pre-DM of strategy 1 was CDRS, while strategy 2 was FPG. The sensitivity of FPG for pre-DM was 41.38%, while that of CDRS was 73.39% (19, 20); thus, strategy 1 has a more vital ability to detect pre-DM than strategy 2. Nevertheless, the specificity of CDRS screening for pre-DM was about 50%, and that of FPG was about 90% (19, 20). Strategy 1 would lead more NGTs to further OGTT tests, thus increasing the costs.

### Sensitivity analysis

The result of the study from a health system perspective was shown in the supplementary and was consistent with the base analysis (Supplementary Table 7). The tornado figure was obtained by one-way sensitivity analysis (Supplementary Results). It showed that the main variables affecting the results were discount rate, cost of OGTT, the misdiagnosis rate of CDRS for NGT, and the transition probability from diagnosed and undiagnosed IGT to T2DM (over 60 years old). Therefore, we again expanded the variation range of these crucial parameters for sensitivity analysis (Supplementary Table 8). When the cost of OGTT was set to triple the original parameters, the ICER of strategy 1 compared with the control group was $1287.42, within the WTP threshold. Furthermore, the results remained stable, even if the misdiagnosis rate of CDRS for NGT, and the transition probability from diagnosed and undiagnosed IGT to T2DM (over 60 years old) increased by 100% and decreased by 50%.

We also conducted a sensitivity analysis of compliance with the screening strategy (Table 3). As the compliance rate decreased, the cost and effectiveness of strategy 1 and strategy 2 decreased. When the screening compliance rate decreased to 1%, compared with the control group, the ICERs of strategy 1 and strategy 2 were $375.31 (95% CI 373.25, 377.36) and $119.81 (95% CI 95.60, 144.02), respectively.

**Table 3:** Sensitivity of clinical and economic data to different compliance rates of screening.

	**Control (95% CI)**	**Strategy 1 (95% CI)**	**Strategy 2 (95% CI)**
**Compliance rate of screening is 80%**			
Cost ($)	6.05 (6.04, 6.05)	41.27 (41.25, 41.29)	27.71 (27.69, 27.73)
Cumulative prevalence of T2DM (%)	63.72 (63.69, 63.74)	52.19 (52.15, 52.22)	55.73 (55.70, 55.76)
Cost per case prevented^a^	–	**305.84 (305.01, 306.68)**	**272.09 (271.08, 273.10)**
Cost per case prevented^b^	–	**385.69 (383.33, 388.05)**	–
**Compliance rate of screening is 60%**			
Cost ($)	6.04 (6.04, 6.05)	35.85 (35.83, 35.87)	22.29 (22.28, 22.31)
Cumulative prevalence of T2DM (%)	63.72 (63.69, 63.74)	54.17 (54.13, 54.20)	57.68 (57.65, 57.72)
Cost per case prevented^a^	–	**312.69 (311.79, 313.59)**	**270.82 (269.59, 272.05)**
Cost per case prevented^b^	–	**388.86 (386.46, 391.27)**	–
**Compliance rate of screening is 40%**			
Cost ($)	6.04 (6.04, 6.05)	30.44 (30.41, 30.45)	16.88 (16.87, 16.90)
Cumulative prevalence of T2DM (%)	63.72 (63.69, 63.74)	56.14 (56.11, 56.17)	59.66 (59.63, 59.69)
Cost per case prevented^a^	–	**322.95 (321.79, 324.12)**	**269.59 (267.91, 271.27)**
Cost per case prevented^b^	–	**389.05 (386.60, 391.50)**	–
**Compliance rate of screening is 20%**			
Cost ($)	6.04 (6.04, 6.05)	25.02 (25.00, 25.04)	11.46 (11.45, 11.47)
Cumulative prevalence of T2DM (%)	63.72 (63.69, 63.74)	58.14 (58.11, 58.17)	61.61 (61.58, 61.64)
Cost per case prevented^a^	–	**341.60 (340.08, 343.12)**	**264.14 (261.13, 267.14)**
Cost per case prevented^b^	–	**395.19 (392.53, 397.85)**	–
**Compliance rate of screening is 1%**			
Cost ($)	6.04 (6.04, 6.05)	19.87 (19.85, 19.89)	6.31 (6.30, 6.31)
Cumulative prevalence of T2DM (%)	63.71 (63.68, 63.74)	59.99 (59.95, 60.01)	63.45 (63.42, 63.48)
Cost per case prevented^a^	–	**375.31 (373.25, 377.36)**	**119.81 (95.60, 144.02)**
Cost per case prevented^b^	–	**394.65 (392.21, 397.09)**	–

a ICER values of the two screening strategies compared with the control group.

b ICER value of comparison between the two screening strategies.

T2DM, type 2 diabetes mellitus. The bold values indicate the ICER values which we are most concerned about.

**Figure 3:**
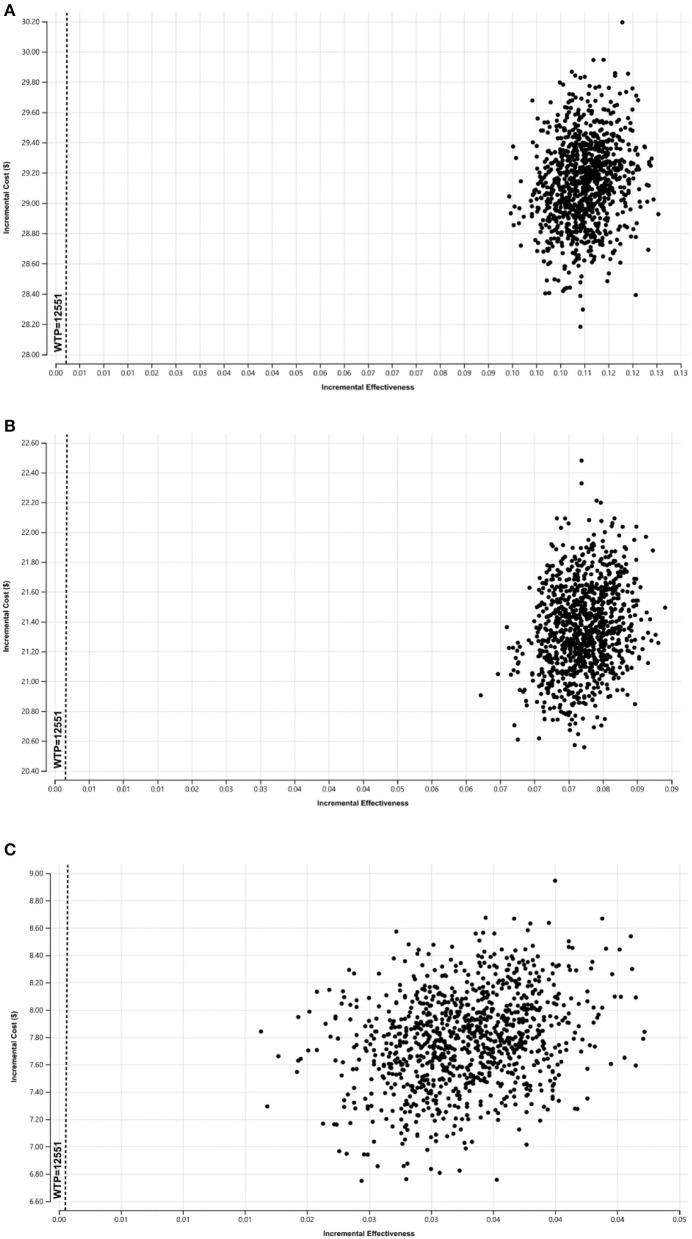
The probabilistic sensitivity results of the ICER in difference of screening strategies, **(A)** is the ICER of strategy 1 compared to the control group, **(B)** is the ICER of strategy 2 compared to the control group, **(C)** is the ICER of screening strategy 1 compared to the strategy 2.

In the probabilistic sensitivity analyses, the probability that these screening strategies were cost-effective compared with the control group was 100%, as was strategy 1 compared to strategy 2 (Figure 3).

## Discussion

Two screening strategies for pre-DM using CDRS were constructed and estimated the cost-effectiveness. Compared with the control group and strategy 2, the ICERs of strategy 1 were $299.67 and $385.89, which were below the WTP threshold. Moreover, the cost a case of T2DM prevented for strategy 2 was $272.23 compared with the control group, suggesting that strategy 2 was also a cost-effective health policy.

The screening strategy 1 for pre-DM was the most cost-effective, and CDRS should be implemented in the whole adult population. CDRS is a questionnaire that includes age, sex, waist circumference, BMI, systolic blood pressure, and diabetes family history (17). Unlike the Finnish Diabetes Risk Score, CDRS can be completed without the guidance of doctors (17, 46). In addition, CDRS has strong accessibility and can be spread to a large number of people in a short time through the media and the Internet (17). Moreover, the diabetes risk scale showed high acceptability by the subjects. The vast majority of subjects indicated that they preferred this screening method rather than the method requiring blood collection (47). Whether the adult population with or without annual health examination, the use of CDRS for pre-DM screening is feasible from the perspective of the measured population. Therefore, we suggest that medical institutions replace the FPG test with CDRS for pre-DM screening; at the same time, self-screening for pre-DM using CDRS is widely promoted among people without an annual health examination.

CDRS is not costly, which contradicts the need for medical institutions to make profits. The strategy 2 that adults without annual health examinations regularly use CDRS to self-evaluate the risk of pre-DM should be considered as an alternative screening strategy. CDRS screening for pre-DM only in adults without annual health examination was still cost-effective compared with the control group. Some surveys showed that the compliance rate of taking risk assessment tools is 37.7–76.4% (48, 49). We found that even if the compliance rate of self-screening using CDRS was reduced to 20%, strategy 2 could still prevent 2.11% of T2DM and was a cost-effective health policy comparable to the control group.

The sensitivity analysis results of all the input indicators in the model showed stable results. Perhaps because i-IGT accounts for half of the pre-DM patients in China, and its sensitivity to lifestyle intervention is more excellent than IFG + IGT, the transition probability of diagnosed and undiagnosed i-IGT to T2DM had a more significant impact on the results (16, 34). We found that even if the transition probability of undiagnosed i-IGT to T2DM decreased by 50% of the original input parameters or the transition probability of diagnosed i-IGT to T2DM increased by 200 % of the initial input parameters, the conclusion was still stable. In addition, it showed that the higher the misdiagnosis rate of CDRS for NGT was, the more people needed to undergo OGTT further, so the cost gradually increased. However, the misdiagnosis rate of CDRS for NGT patients reached 89.48%, double the initial input parameter, strategy 1 and strategy 2 were still cost-effective compared to the control group. Similar to previous studies, we found maintaining higher screening compliance can achieve higher cost and effectiveness (50), while ICER values vary little among different strategies. The conclusions remain stable, even if the screening compliance rate was reduced to 1%.

To the best of our knowledge, this study is the first to construct CDRS screening strategies, including screening and lifestyle intervention for pre-DM, and evaluate the long-term impact of these strategies on the course of pre-DM and economics in China. Moreover, studies have shown that different types of pre-DM respond differently to the same intervention (51). However, existing studies in China ignored i-IFG status or did not distinguish IGT and IFG + IGT, which may underestimate the number of patients with pre-DM (50, 52). In contrast, we considered three pre-DM states, including i-IFG, i-IGT, and IFG + IGT, and more comprehensively. In addition, we set the control group closer to reality according to the current practice of finding pre-DM.

There were also a few limitations of our study. First, as we ran the model for a long horizon, the screened population aged with each cycle of the model, thus, when possible, we set the model parameters as time-dependent parameters, like the transition probabilities from pre-DM to T2DM. However, due to the difficulty of obtaining data, some parameters were assumed to maintain invariable even time changes, such as transition probabilities from NGT to pre-DM. We also conducted several sensitivity analyses for these parameters and showed the robustness of our results. Secondly, due to the lack of data, some parameters in the model were not in the context of China. We selected the data from Japan, a country in Asia, such as the transfer rate of i-IFG to T2DM (35). Sensitivity analysis also showed that the results were stable and credible.

## Conclusion

CDRS screening for pre-DM in all adults was the most cost-effective health policy. We suggest that medical institutions replace FPG with CDRS for pre-DM screening; at the same time, self-screening for pre-DM using CDRS is widely promoted among adults without an annual health examination. Considering there were still some disputes about how CDRS is included in the health examination projects, regularly using CDRS to self-screen for pre-DM in adults without annual health examination should be considered as an alternative screening strategy. The present study can provide new research clues and directions for evaluating and applying such a kind of screening instrument for pre-DM, especially in other developing countries, to promote the prevention of T2DM.

## Data availability statement

The original contributions presented in the study are included in the article/Supplementary material, further inquiries can be directed to the corresponding author.

## Author contributions

JH conceptualized the study, collected data, conducted analysis, and wrote the initial draft. QY and CZ contributed to the analysis, interpretation of results, and the final drafting of the manuscript. YL, YS, BZ, MH, JZ, NZ, JP, and ZL contributed to the final drafting of the manuscript. All authors prepared the manuscript and approved the final version for submission.

## Funding

This study was supported by funding from the National Natural Science foundation of China (Grant No. 82173618).

## Conflict of interest

The authors declare that the research was conducted in the absence of any commercial or financial relationships that could be construed as a potential conflict of interest.

## Publisher's note

All claims expressed in this article are solely those of the authors and do not necessarily represent those of their affiliated organizations, or those of the publisher, the editors and the reviewers. Any product that may be evaluated in this article, or claim that may be made by its manufacturer, is not guaranteed or endorsed by the publisher.

## Abbreviations

CDRS: Chinese Diabetes Risk Score
FPG: Fasting glucose test
ICER: Incremental cost-effectiveness ratio
I-IFG: Isolated impaired fasting glucose
IFG + IGT: Impaired fasting glucose and impaired glucose tolerance
I-IGT: Isolated impaired glucose tolerance
NGT: Normal glucose tolerance
Pre-DM: Pre-diabetes mellitus
T2DM: Type 2 diabetes mellitus
WTP: willingness to pay.
