# Supplementary material for: Cost-effectiveness of two screening strategies based on Chinese diabetes risk score for pre-diabetes in China
Source: Front Public Health. 2022 Nov 30;10:1018084. doi: 10.3389/fpubh.2022.1018084 (PMC9747942; doi:10.3389/fpubh.2022.1018084)
Supplement: Supplementary file 1 [file Data_Sheet_1.docx]

**Supplementary Material**

# **Methods**

We set different distributions for different variable types for probabilistic sensitivity analysis, and the settings were as follows:

(Ⅰ) For dichotomous variables, the beta distribution is suitable if there were original data or the number of cases and sample size reported in previous literature. The probability density function was as follows (1):

$$f_{(x;\alpha;\beta)}=\frac{\Gamma\left( \alpha+\beta\right)}{\Gamma\left( \alpha\right)\Gamma\left( \beta\right)}x^{\alpha-1}{(1-x)}^{\beta-1}$$

α and β are two critical parameters of the beta distribution; α represent the number of events occurring, and β represent the number of events not happening. Parameters fitting the beta distribution in this study are shown in Table S2.

(Ⅱ)Health costs are usually distributed skewness. In this study, it was assumed that costs follow a lognormal distribution. The logarithmic mean was taken as the mean, and the standard deviation was 10% of the mean. The probability density function was as follows (2):

$$f(x)=\frac{1}{x\sigma\sqrt{2\pi}}e^{(\frac{-(\ln(x)-\mu)^{2}}{2\sigma^{2}})}$$

Where μ and σ are logarithmic mean and logarithmic standard deviation, respectively, parameters fitting lognormal distribution in this study are shown in Table S3.

(Ⅲ) Other variables were fitted as uniform distribution because specific distribution parameters could not be calculated from previous literature or actual situations. The parameters fitting uniform distribution in this study are shown in Table S4.

# **Results**

The tornado figure was obtained by one-way sensitivity analysis (Figure S1). As shown in the figure, each variable would generate a horizontal bar. The abscissa represented the incremental cost-effectiveness ratio (ICER) corresponding to different parameter settings. The color change of the horizontal bar represented the influence of the parameter on the result direction. The left side of the horizontal bar was gray, and the right side was black, indicating that ICER increased with increased parameters. In Fig.1a, the higher the cost of OGTT was, the higher the cost that strategy 1 needed to pay compared with the control group, but the effect would not be affected, so the ICER value would increase. The vertical line in the figure represents the mean value of ICER obtained by model operation. The mean ICERs in the three graphs were within the willingness-to-pay (WTP) threshold, indicating that the conclusion remained stable.

In this figure, the length change of the horizontal bar represented its influence on the model result index. All parameters were sorted from top to bottom according to their influence on the model. For example, the critical parameters affecting the ICER value of strategy 2 compared with the control group by the degree of influence were the discounting rate, the transition probability from diagnosed isolated impaired glucose tolerance (i-IGT) to type 2 diabetes mellitus (T2DM) (over 60 years old), the misdiagnosis rate of Chinese Diabetes Risk Score (CDRS) for normal glucose tolerance (NGT), the cost of OGTT and the transition probability from diagnosed isolated impaired fasting glucose (i-IFG) to T2DM (more than 60 years old). However, whatever the discounting rate changed, the results were still stable.

# **References**

1. Pasta DJ, Taylor JL, Henning JM. Probabilistic sensitivity analysis incorporating the bootstrap: an example comparing treatments for the eradication of Helicobacter pylori. Med Decis Making. 1999 Jul-Sep;19(3):353-63. Epub 1999/07/29. doi:10.1177/0272989X9901900314. Cited in: Pubmed; PMID 10424842.

2. Decision analytic modelling in the economic evaluation of health technologies. A consensus statement. Consensus Conference on Guidelines on Economic Modelling in Health Technology Assessment. Pharmacoeconomics. 2000 May;17(5):443-4. Epub 2000/09/08. doi:10.2165/00019053-200017050-00003. Cited in: Pubmed; PMID 10977386.

3. Wang L, Gao P, Zhang M, Huang Z, Zhang D, Deng Q, Li Y, Zhao Z, Qin X, Jin D, Zhou M, Tang X, Hu Y, Wang L. Prevalence and Ethnic Pattern of Diabetes and Prediabetes in China in 2013. JAMA. 2017 Jun 27;317(24):2515-2523. Epub 2017/06/28. doi:10.1001/jama.2017.7596. Cited in: Pubmed; PMID 28655017.

4. Ye Z, Cong L, Ding G, Yu M, Zhang X, Hu R, Wu J, Fang L, Wang H, Zhang J, He Q, Su D, Zhao M, Wang L, Gong W, Xiao Y, Liang M, Pan J. Optimal cut-off points for two-step strategy in screening of undiagnosed diabetes: a population-based study in China. PLoS One. 2014;9(3):e87690. Epub 2014/03/13. doi:10.1371/journal.pone.0087690. Cited in: Pubmed; PMID 24609110.

5. Mao T, Chen J, Guo H, Qu C, He C, Xu X, Yang G, Zhen S, Li X. The Efficacy of New Chinese Diabetes Risk Score in Screening Undiagnosed Type 2 Diabetes and Prediabetes: A Community-Based Cross-Sectional Study in Eastern China. J Diabetes Res. 2020;2020:7463082. Epub 2020/05/15. doi:10.1155/2020/7463082. Cited in: Pubmed; PMID 32405505.

6. Zhang F, Wan Q, Cao H, Tang L, Li D, Lu Q, Yan Z, Li J, Yang Q, Zhang Y, Tong N. Identical anthropometric characteristics of impaired fasting glucose combined with impaired glucose tolerance and newly diagnosed type 2 diabetes: anthropometric indicators to predict hyperglycaemia in a community-based prospective cohort study in southwest China. BMJ Open. 2018 May 9;8(5):e019735. Epub 2018/05/11. doi:10.1136/bmjopen-2017-019735. Cited in: Pubmed; PMID 29743321.

7. Zhang Y, Sun J, Pang Z, Gao W, Sintonen H, Kapur A, Qiao Q. Evaluation of two screening methods for undiagnosed diabetes in China: an cost-effectiveness study. Prim Care Diabetes. 2013 Dec;7(4):275-82. Epub 2013/09/12. doi:10.1016/j.pcd.2013.08.003. Cited in: Pubmed; PMID 24021478.

8. association Cmd. White Paper on the Practice of Chinese Doctors. 2015. Available from: <http://www.cmda.net/>.

9. nongping F. The cost-effectivness of standardized community diabetes management [master]: Chinese Center for Disease Control and Prevention; 2009. Cnki.

10. Statistics NBo. Household income and consumption expenditure in 2021. <http://www.stats.gov.cn/xxgk/sjfb/zxfb2020/202201/t20220117_1826442.html2022> 2022-01-17.

11. liuxin W. Blue book of health management—— Annual report on development of health management and health industry in China (2018). No.1 ed. Beijing: Social sciences academic press(China); 2019. ISBN: 978-7-5201-4531-2.

12. Mao T, Qu C, He C. Analysis of detection rates by different diagnostic criterias for newly diagnosed diabetes and prediabetes among community residents aged 18 to 65 years in Jiangsu province. Jiangsu Provincial Center for Disease Control and Prevention. 2019;30(04):401-404+413. doi:10.13668/j.issn.1006-9070.2019.04.013.

13. Xu L, Jiang CQ, Lam TH, Cheng KK, Yue XJ, Lin JM, Zhang WS, Thomas GN. Impact of impaired fasting glucose and impaired glucose tolerance on arterial stiffness in an older Chinese population: the Guangzhou Biobank Cohort Study-CVD. Metabolism. 2010 Mar;59(3):367-72. Epub 2009/10/16. doi:10.1016/j.metabol.2009.08.004. Cited in: Pubmed; PMID 19828159.

14. Chen G, Shi L, Cai L, Lin W, Huang H, Liang J, Li L, Lin L, Tang K, Chen L, Lu J, Bi Y, Wang W, Ning G, Wen J. Comparison of Insulin Resistance and beta-Cell Dysfunction Between the Young and the Elderly in Normal Glucose Tolerance and Prediabetes Population: A Prospective Study. Horm Metab Res. 2017 Feb;49(2):135-141. Epub 2016/07/28. doi:10.1055/s-0042-111325. Cited in: Pubmed; PMID 27459384.

15. Liu L, Guan X, Yuan Z, Zhao M, Li Q, Zhang X, Zhang H, Zheng D, Xu J, Gao L, Guan Q, Zhao J, The Reaction Study G. Different Contributions of Dyslipidemia and Obesity to the Natural History of Type 2 Diabetes: 3-Year Cohort Study in China. J Diabetes Res. 2019;2019:4328975. Epub 2019/04/06. doi:10.1155/2019/4328975. Cited in: Pubmed; PMID 30949514.

16. Saito T, Watanabe M, Nishida J, Izumi T, Omura M, Takagi T, Fukunaga R, Bandai Y, Tajima N, Nakamura Y, Ito M, Zensharen Study for Prevention of Lifestyle Diseases G. Lifestyle modification and prevention of type 2 diabetes in overweight Japanese with impaired fasting glucose levels: a randomized controlled trial. Arch Intern Med. 2011 Aug 8;171(15):1352-60. Epub 2011/08/10. doi:10.1001/archinternmed.2011.275. Cited in: Pubmed; PMID 21824948.

17. Gong Q, Zhang P, Wang J, Gregg EW, Cheng YJ, Li G, Bennett PH, Da Qing Diabetes Prevention Outcome Study G. Efficacy of lifestyle intervention in adults with impaired glucose tolerance with and without impaired fasting plasma glucose: A post hoc analysis of Da Qing Diabetes Prevention Outcome Study. Diabetes Obes Metab. 2021 Oct;23(10):2385-2394. Epub 2021/07/03. doi:10.1111/dom.14481. Cited in: Pubmed; PMID 34212465.

18. Qian Q, Li X, Huang X, Fu M, Meng Z, Chen M, Feng B. Glucose metabolism among residents in Shanghai: natural outcome of a 5-year follow-up study. J Endocrinol Invest. 2012 May;35(5):453-8. Epub 2011/07/09. doi:10.3275/7854. Cited in: Pubmed; PMID 21738002.

## **Table S1.** Chinese Diabetes Risk Score

| Index | Scores | Index | | Scores |
| --- | --- | --- | --- | --- |
| Age (years) |  | BMI（kg/m2) | |  |
| 20~24 | 0 | <22 | | 0 |
| 25~34 | 4 | 22.0~23.9 | | 1 |
| 35~39 | 8 | 24.0~29.9 | | 3 |
| 40~44 | 11 | ≥30.0 | | 5 |
| 45~49 | 12 | Waist(cm) | |  |
| 50~54 | 13 | Male<75, female<70.0 | | 0 |
| 55~59 | 15 | Male 75.0~79.9, female 70.0~74.9 | | 3 |
| 60~64 | 16 | Male 80.0~84.9, female 75.0~79.9 | | 5 |
| 65~74 | 18 | Male 85.0~89.9, female 80.0~84.9 | | 7 |
| Systolic pressure(mmHg) |  | Male 90.0~94.9, female 85.0~89.9 | | 8 |
| <110 | 0 | Male≥95.0, female≥90.0 |  | 10 |
| 110~119 | 1 | Family history of T2DM | |  |
| 120~129 | 3 | No | | 0 |
| 130~139 | 6 | Yes | | 6 |
| 140~149 | 7 | Gender | |  |
| 150~159 | 8 | Female | | 0 |
| ≥160 | 10 | Male | | 2 |

T2DM, type 2 diabetes mellitus

## **Table S2.** Parameter distributions of beta distributions in probabilistic sensitivity analysis

| Parameters | Expected values | α | β | reference |
| --- | --- | --- | --- | --- |
| Prevalence of undiagnosed T2DM ^a^ |  |  |  |  |
| <40 | 4.56% | 1596 | 33409 | (3) |
| 40-60 | 8.22% | 6652 | 74363 | (3) |
| >60 | 12.50% | 5648 | 39534 | (3) |
| Sensitivity of FPG for i-IFG | 46.09% | 165 | 193 | (4) |
| Sensitivity of FPG for T2DM | 73.42% | 511 | 185 | (4) |
| Sensitivity of FPG for IFG+IGT | 69.74% | 136 | 59 | (4) |
| Sensitivity of CDRS for Pre-DM | 73.39% | 943 | 342 | (5) |
| Misdiagnosis rate of CDRS for NGT | 44.74% | 2635 | 3255 | (5) |
| Transition rate of NGT to i-IFG | 2.04% | 159 | 1726 | (6) |
| Transition rate of NGT to i-IGT | 6.46% | 342 | 1543 | (6) |
| Transition rate of NGT to IFG+IGT | 2.76% | 152 | 1733 | (6) |

^a^ The input parameter varied by age.

T2DM, type 2 diabetes mellitus; FPG, fasting glucose test; I-IFG, isolated impaired fasting glucose; I-IGT, isolated impaired glucose tolerance; IFG+IGT, impaired fasting glucose and impaired glucose tolerance; NGT, normal glucose tolerance; Pre-DM, pre-diabetes mellitus; CDRS, Chinese Diabetes Risk Score.

## **Table S3.** Parameter distributions of lognormal distributions in probabilistic sensitivity analysis

|  | values（$）^a^ | μ | σ | reference |
| --- | --- | --- | --- | --- |
| FPG | 5.49 | 1.70 | 0.17 | (4, 7-10) |
| OGTT | 13.36 | 2.59 | 0.26 | (4, 7-10) |
| Life intervention | 26.66 | 3.28 | 0.33 | (4, 7-10) |

^a^ All cost data were shown in the 2021 US dollar ($1= ¥ 6.45).

FPG, fasting glucose test; OGTT, oral glucose tolerance test.

## **Table S4.** Parameter distributions of uniform distributions in the probabilistic sensitivity analysis

| Parameters | Expected values | Ranges | reference |
| --- | --- | --- | --- |
| **Demographic variables** |  |  |  |
| Proportion of Physical examination | 42.00% | 31.5%-52.50% | (11) |
| Prevalence of undetected i-IFG ^a^ | | | |
| <40 | 17.28% | 12.96%-21.60% | (3, 12, 13) |
| 40-60 | 10.18% | 7.64%-12.73% | (3, 12, 13) |
| >60 | 13.94% | 10.45%-17.42% | (3, 12, 13) |
| Prevalence of undetected i-IGT ^a^ |  |  |  |
| <40 | 8.21% | 6.16%-10.26% | (3, 12, 13) |
| 40-60 | 20.51% | 15.38%-25.64% | (3, 12, 13) |
| >60 | 22.59% | 16.94%-28.23% | (3, 12, 13) |
| Prevalence of undetected IFG+IGT ^a^ |  |  |  |
| <40 | 3.72% | 2.79%-4.64% | (3, 12, 13) |
| 40-60 | 10.93% | 8.20%-13.66% | (3, 12, 13) |
| >60 | 13.70% | 10.27%-17.12% | (3, 12, 13) |
| **Test efficiency** |  |  |  |
| Sensitivity of CDRS for T2DM | 87.60% | 65.7%-1.00 | (7) |
| **Transition probabilities** |  |  |  |
| Undiagnosed i-IFG to T2DM ^a^ |  |  |  |
| <40 | 7.79% | 5.84%-9.74% | (14) |
| 40-50 | 2.90% | 2.48%-4.92% | (14, 15) |
| 50-60 | 2.98% | 1.35%-2.25% | (14, 15) |
| >60 | 3.59% | 2.69%-4.49% | (14) |
| Diagnosed i-IFG to T2DM ^a^ | | |  |
| <40 | 9.11% | 3.90%-21.34% | (14, 16) |
| 40-50 | 3.39% | 1.45%-7.93% | (14-16) |
| 50-60 | 3.49% | 1.49%-8.17% | (14-16) |
| >60 | 4.20% | 1.80%-9.84% | (14, 16) |
| Undiagnosed i-IGT to T2DM ^a^ | | |  |
| <40 | 10.91% | 8.18%-13.64% | (14) |
| 40-50 | 6.38% | 5.46%-10.84% | (14, 15) |
| 50-60 | 6.57% | 4.47%-9.09% | (14, 15) |
| >60 | 7.91% | 5.93%-9.89% | (14) |
| Diagnosed i-IGT to T2DM ^a^ | | |  |
| <40 | 5.89% | 3.93%-8.84% | (14, 17) |
| 40-50 | 3.44% | 2.30%-5.17% | (14, 15, 17) |
| 50-60 | 3.55% | 2.37%-5.32% | (14, 15, 17) |
| >60 | 4.27% | 2.85%-6.41% | (14, 17) |
| Undiagnosed IGT+IFG to T2DM ^a^ | | |  |
| <40 | 8.54% | 6.41%-10.68% | (14) |
| 40-50 | 11.11% | 9.05%-18.88% | (14, 15) |
| 50-60 | 11.45% | 7.79%-15.84% | (14, 15) |
| >60 | 13.78% | 10.34%-17.23% | (14) |
| Diagnosed IGT+IFG to T2DM ^a^ | | |  |
| <40 | 4.27% | 3.07%-5.89% | (14, 17) |
| 40-50 | 5.56% | 4.00%-7.67% | (14, 15, 17) |
| 50-60 | 5.72% | 4.12%-7.90% | (14, 15, 17) |
| >60 | 6.89% | 4.96%-9.51% | (14, 17) |
| i-IFG to NGT | 6.89% | 5.16%-8.61% | (18) |
| i-IGT to NGT | 8.83% | 6.62%-11.03% | (18) |
| IGT+IFG to NGT | 5.34% | 4.01%-6.68% | (18) |

^a^ These input parameters were varied by age.

T2DM, type 2 diabetes mellitus; FPG, fasting glucose test; I-IFG, isolated impaired fasting glucose; I-IGT, isolated impaired glucose tolerance; IFG+IGT, impaired fasting glucose and impaired glucose tolerance; NGT, normal glucose tolerance; OGTT, oral glucose tolerance test; Pre-DM, pre-diabetes mellitus; CDRS, Chinese Diabetes Risk Score.

## **Table S5.** The detailed list of screening and lifestyle intervention costs

| Parameters | Cost（95% CI）^a^ | Reference |
| --- | --- | --- |
| **Cost of FPG/Event** | **5.49（3.64-7.97）** |  |
| Direct medical costs/ Event | 1.19（1.05-1.95） |  |
| Laboratory tests | 0.43 | (4) |
| Medical personnel time | 0.76（0.62-1.52） | (4, 7, 8) |
| Direct non-medical costs / Event | 3.40（1.69-5.12） | (9) |
| Indirect costs / Event | 0.9 | (7, 10) |
| **Cost of OGTT/ Event** | **13.36（11.62-15.82）** |  |
| Direct medical costs/ Event | 1.83（1.80-2.57） |  |
| Laboratory tests | 1.05 | (4) |
| Medical personnel time | 0.78（0.75-1.52） | (4, 7, 8) |
| Direct non-medical costs / Event | 3.40（1.69-5.12） | (9) |
| Indirect costs / Event | 8.13 | (7, 10) |
| **Cost of Lifestyle Intervention/Event** | **26.66（22.06-31.98）** |  |
| Direct medical costs/ Event | 12.41（9.52-16.01） |  |
| Direct medical costs of OGTT | 1.83（1.80-2.57） | (4, 7, 8) |
| Medical personnel time | 10.58(7.72-13.44) | (4) |
| Direct non-medical costs / Event | 3.40（1.69-5.12） | (4) |
| Indirect costs / Event | 10.85 | (10) |

^a^ All cost data were shown in the 2021 US dollar ($1=¥6.45).

FPG, fasting glucose test; OGTT, oral glucose tolerance test.

## **Table S6.** The costs setting from the health system perspective

| Parameters | Cost（95% CI）^a^ | Reference |
| --- | --- | --- |
| **Cost of FPG/Event** | **1.19（1.05-1.95）** |  |
| Direct medical costs/ Event | 1.19（1.05-1.95） |  |
| Laboratory tests | 0.43 | (4) |
| Medical personnel time | 0.76（0.62-1.52） | (4, 7, 8) |
| **Cost of OGTT/ Event** | **1.83（1.80-2.57）** |  |
| Direct medical costs/ Event | 1.83（1.80-2.57） |  |
| Laboratory tests | 1.05 | (4) |
| Medical personnel time | 0.78（0.75-1.52） | (4, 7, 8) |
| **Cost of Lifestyle Intervention/Event** | **12.41（9.52-16.01）** |  |
| Direct medical costs/ Event | 12.41（9.52-16.01） |  |
| Direct medical costs of OGTT | 1.83（1.80-2.57） | (4, 7, 8) |
| Medical personnel time | 10.58(7.72-13.44) | (4) |

^a^ All cost data were shown in the 2021 US dollar ($1=¥6.45).

FPG, fasting glucose test; OGTT, oral glucose tolerance test.

## **Table S7.** Clinical and economic results for different screening strategies from health system perspective

|  | Control (95% *CI*) | Strategy 1 (95% *CI*) | Strategy 2 (95% *CI*) |
| --- | --- | --- | --- |
| Cost ($) | 1.01(1.01,1.01) | 6.40(6.39,6.40) | 4.72(4.72,4.72) |
| Cumulative prevalence of T2DM (%) | 63.72(63.69,63.74) | 50.14(50.10,50.17) | 53.75(53.71,53.78) |
| Cost per case prevented ^a^ | - | **39.70(39.60,39.81)** | **37.23(37.13,37.33)** |
| Cost per case prevented ^b^ | - | **47.83(47.31,48.36)** | **-** |

^a^ ICER values of the two screening strategies compared with the control group

^b^ ICER value of comparison between the two screening strategies

T2DM, type 2 diabetes mellitus.

## **Table S8.** Sensitivity analyses of clinical and economic data to some input parameters

|  | | Control | Strategy 1 | Strategy 2 |
| --- | --- | --- | --- | --- |
| **Cost of OGTT (triple)** |  |  |  |  |
| Cost ($) | 13.52 | 140.11 | 94.81 |  |
| Cumulative prevalence of T2DM (%) | 63.72 | 50.15 | 53.76 |  |
| Cost per case prevented ^a^ | - | **934.21** | **818.27** |  |
| Cost per case prevented ^b^ | - | **1287.42** | **-** |  |
| **Undiagnosed i-IGT to T2DM (>60) ^c^** |  |  |  |  |
| Cost ($) | 5.85/6.15 | 46.84/46.80 | 33.03/33.30 |  |
| Cumulative prevalence of T2DM (%) | 58.13/69.68 | 49.69/50.61 | 50.63/56.60 |  |
| Cost per case prevented ^a^ | - | **485.66/213.16** | **362.42/207.53** |  |
| Cost per case prevented ^b^ | - | **1469.12/225.38** | **-** |  |
| **Diagnosed i-IGT to T2DM (>60) ^c^** |  |  |  |  |
| Cost ($) | 5.93/2.92 | 46.82/45.86 | 33.47/32.91 |  |
| Cumulative prevalence of T2DM (%) | 64.26/66.00 | 44.36/59.32 | 49.98/60.67 |  |
| Cost per case prevented ^a^ | - | **205.49/597.89** | **192.85/506.36** |  |
| Cost per case prevented ^b^ | - | **237.59/959.26** | **-** |  |
| **Misdiagnosis rate of CDRS for NGT ^c^** |  |  |  |  |
| Cost ($) | 6.01/6.01 | 30.12/80.78 | 23.38/51.75 |  |
| Cumulative prevalence of T2DM (%) | 63.62/63.62 | 49.51/49.51 | 53.95/53.95 |  |
| Cost per case prevented ^a^ | - | **170.83/529.87** | **179.58/473.03** |  |
| Cost per case prevented ^b^ | - | **151.77/653.64** | **-** |  |

^a^ ICER values of the two screening strategies compared with the control group.

^b^ ICER value of comparison between the two screening strategies.

^c^ the result of setting the parameter to 50% of the original input parameter / the result of setting the parameter to 200% of the original input parameter.

OGTT, oral glucose tolerance test; T2DM, type 2 diabetes mellitus; I-IGT, isolated impaired glucose tolerance; NGT, normal glucose tolerance.


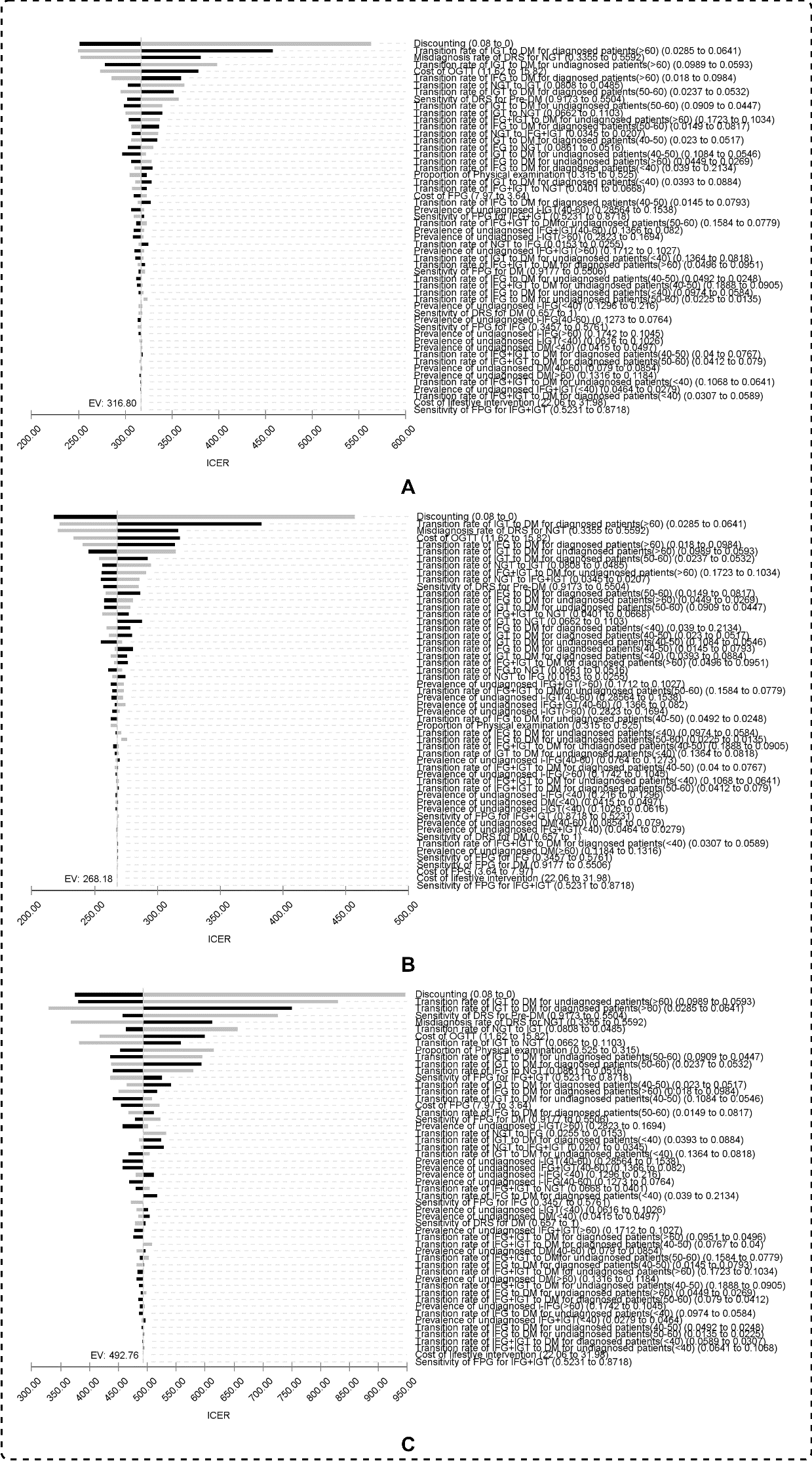


**Figure S1.** The tornado diagram of the incremental cost-effectiveness difference of screening strategies, **(A)** is tornado diagram of the strategy 1 compared to control group, **(B)** is tornado diagram of the strategy 2 compared to control group, **(C)** is tornado diagram of the screening strategy 1 compared to strategy 2.

T2DM, type 2 diabetes mellitus; FPG, fasting glucose test; I-IFG, isolated impaired fasting glucose; I-IGT, isolated impaired glucose tolerance; IFG+IGT, impaired fasting glucose and impaired glucose tolerance; NGT, normal glucose tolerance; OGTT, oral glucose tolerance test; Pre-DM, pre-diabetes mellitus; CDRS, Chinese Diabetes Risk Score.
